# A qualitative comparison of the nutrition care experiences of carers supporting patients with head and neck cancer throughout surgery and radiation treatment and survivorship

**DOI:** 10.1007/s00520-022-07348-0

**Published:** 2022-09-16

**Authors:** Joanne Hiatt, Adrienne Young, Teresa Brown, Merrilyn Banks, Bronwyn Segon, Judith Bauer

**Affiliations:** 1 School of Human Movement and Nutrition Sciences, University of Queensland, St Lucia, Australia; 2 Department of Nutrition and Dietetics, Royal Brisbane and Women’s Hospital, Level 2, Dr James Mayne Building, Butterfield Street, 4029 Herston, QLD Australia

**Keywords:** Head and neck neoplasms, Qualitative research, Caregivers, Nutritional support, Radiation oncology, Surgery

## Abstract

**Purpose:**

To understand and compare the nutrition care experiences of carers supporting patients throughout surgery and radiation treatment for head and neck cancer (HNC) to inform changes to service delivery in the inpatient and outpatient setting to ensure carers needs in their supportive role throughout the treatment and survivorship period are met.

**Methods:**

As part of a larger study, narrative interviews were completed with fourteen carers of patients diagnosed with HNC at 2 weeks, 3 months and 12 months post-treatment completion. Reflexive thematic analysis was used to interpret and understand differences in carer experiences of nutrition care between surgery and radiation treatment.

**Results:**

Two main themes across each treatment modality were identified: (1) access to information and support from healthcare professionals and (2) adjustment to the physical and psychological impact of treatment.

**Conclusion:**

This study highlights the increasing need to ensure carers are included in the provision of nutrition information and support to patients throughout and beyond their treatment trajectory. Having structured support available to patients and carers throughout radiation treatment meant that carer needs were reduced. However, without the opportunity for structured support in the inpatient setting, many carers expressed high care needs in supporting patients in the post-surgical phase.

**Implications for cancer survivors:**

Providing carers with access to structured support for nutrition care in the inpatient and outpatient setting can reduce their supportive care needs throughout the treatment and survivorship period.

**Supplementary Information:**

The online version contains supplementary material available at 10.1007/s00520-022-07348-0.

## Introduction

Worldwide, head and neck cancer (HNC) is the sixth most common cancer, with incidence rates estimated to increase by 30% in the next 10 years [1, 2]. Surgery, radiation, and chemotherapy are the main treatment modalities for HNC, with many patients needing multimodality treatments [3]. Side effects of treatment include dysphagia, xerostomia, dysgeusia, odynophagia and anatomical changes impacting mastication, all of which may impact eating and drinking [4]. Consequently, nutrition care plays a critical role in the provision of best practice care for patients diagnosed with HNC [5, 6]. This includes ensuring patients have access to dietetic support prior to, throughout and beyond the treatment period. However, outside of the hospital environment, a large proportion of care is provided by informal carers, usually family members or friends [7].

Carers play a crucial role in patient wellbeing, providing emotional and practical support including, food preparation and tube feeding assistance [8, 9]. Patients with HNC are at higher risk of suicide and depression in comparison to the general population [10]; however, studies have shown that carers of patients with HNC experience higher levels of psychological stress than patients, reporting a lack of training and feeling unprepared in managing the complexity of the supportive care role contributing to this stress [11, 12]. With increasing recognition of the importance of the role that the carer plays in supporting the patient throughout cancer treatment and survivorship, studies have sought to further understand the experience of carers in providing support for patients with HNC to further understand their supportive care needs [12]. However, the majority of literature has focused on the carers of patients having radiation treatment, where access to information and support from healthcare professionals is mostly provided in an outpatient setting [13, 14]. It is recognised that further research is needed in understanding the experiences and needs of informal caregivers in cancer surgery, where access to information and support throughout the treatment is provided in an inpatient setting, with follow-up provided in outpatient clinics [7]. Therefore, the aim of this study was to explore and understand differences in the carer experience of nutrition care throughout surgery and radiation/chemoradiation treatment to identify changes to service delivery in the inpatient and outpatient setting to ensure carer needs in their supportive care role are met throughout the treatment and survivorship period.

## Methods

### Study design

A qualitative inductive approach was adopted to explore carer experience of nutrition care following HNC treatment. This study is part of a larger qualitative study using narrative interviews to explore patient and carer experience of nutrition care at four time points from diagnosis through to 1-year post-treatment completion [15]. Interviews were conducted by the principal investigator (JH), a female clinical dietitian with 4 years of experience working in HNC. Training in qualitative research including interview skills, data analysis and interpretation was undertaken by the principal investigator as part of their doctoral education. The interviewer was not directly involved in the clinical care of the patient, and not known to study participants. Reflexive thematic analysis [16] based on Gadamerian hermeneutic inquiry [17] was used for this study to identify and examine themes from interview transcripts to describe and compare carer experiences of nutrition care between different modalities at 2 weeks, 3 months and 12 months post-treatment completion.

### Setting

In the setting for this study, patients diagnosed with HNC attend a multidisciplinary clinic at a tertiary hospital, which their carer may also attend. At this clinic, their treatment plan is developed, which typically includes surgery (followed by post-operative radiation therapy (PORT) in some cases) or radiation therapy (with concurrent chemotherapy in some cases). Aligning with local hospital protocol, patients screened as being at risk of malnutrition are referred for dietetic intervention prior to treatment. Those identified at future risk of nutritional decline during chemoradiotherapy are recommended prophylactic gastrostomy tube placement for nutrition support if required. All patients undergoing radiation treatment are booked in to attend a 2-h group education session run by healthcare professionals including a dentist, speech pathologist, dietitian, social worker, physiotherapist and psychologist to provide an overview of their support needs throughout treatment. Patients undergoing radiation treatment are seen weekly by the dietitian throughout treatment and are followed up fortnightly for a minimum of 6 weeks after treatment completion and then clinically as required. Patients undergoing surgery requiring enteral feeding and those identified as needing support with their oral intake are seen on the ward by the dietitian with follow-up care scheduled weekly to fortnightly as needed. Carers are welcome and encouraged to attend all appointments organised in the joint speech pathology and dietetic clinics for the patient.

### Participants

Participants were recruited from a multidisciplinary HNC clinic at a large tertiary/quaternary hospital in Queensland, Australia. Carer participants were eligible to participate if they were caring for someone with their first diagnosis of HNC and suitable for curative intent treatment. Carers were purposively selected to capture their caregiving role across a range of treatment modalities. Based on previous qualitative studies, the target sample size of carer participants was 15 [13, 18]. All participants were fluent in English, above the age of 18 and able to provide informed consent. All participants were approached face to face and provided with verbal and written information about the study. Consent was obtained one week later by telephone.

### Procedures

Interview transcripts collected as part of the larger study were used to explore carer experiences of nutrition care across and between different treatment modalities for HNC prospectively between June 2018 and April 2020, with interviews at four timepoints (prior to treatment commencing, and then 2 weeks, 3 months and 12 months post-treatment completion). Interviews lasted between 10 and 60 min. Participants were given the option of participating in telephone or face-to-face interviews to allow ease of participation for patients and carers living in regional and rural locations as all interviews were conducted outside of the treatment period. All interviews were conducted by the principal investigator, and the same procedure was used at each interview time point. Prior to each interview commencing, participants were given a broad definition of nutrition care:

“Nutrition care refers to any aspect of care relatable to nutrition. This includes interactions with healthcare professionals, friends and family or any other sources that have provided you with information/care relating to nutrition”

After being provided this definition, participants were asked to describe their experiences. Prompts were used to seek further elaboration around, health and nutrition beliefs, impact on lifestyle, and the emotional and physical impact on participants. As narrative interviews were conducted, a set interview guide was not used. Carer participants were aware that the goals of the research study were to identify improvements to nutrition care services.

Interviews were audio recorded and transcribed verbatim by the principal investigator.

### Data analysis

As the aim of this study was to explore differences in the carer experience of nutrition care between the phases of surgery management and radiation/chemoradiation treatment, interviews conducted prior to treatment commencing were excluded from this study. The transcribed text of interviews conducted at 2 weeks, 3 months and 12 months post-treatment completion was analysed using the six steps of thematic analysis as outlined by Braun and Clarke which included (1) familiarisation with data, (2) generating initial codes, (3) searching for themes, (4) reviewing themes, (5) defining and naming themes and (6) reporting on themes [19]. Consensus was established through regular meetings with a second researcher where codes and emerging themes were reviewed against the dataset to ensure interpretation accuracy. For carers of patients having surgery and PORT, carer experiences of each treatment modality were analysed separately. The Consolidated Criteria for Reporting Qualitative Research (COREQ) checklist was used to ensure comprehensive reporting.

## Results

### Participant demographics

**Fig. 1:**
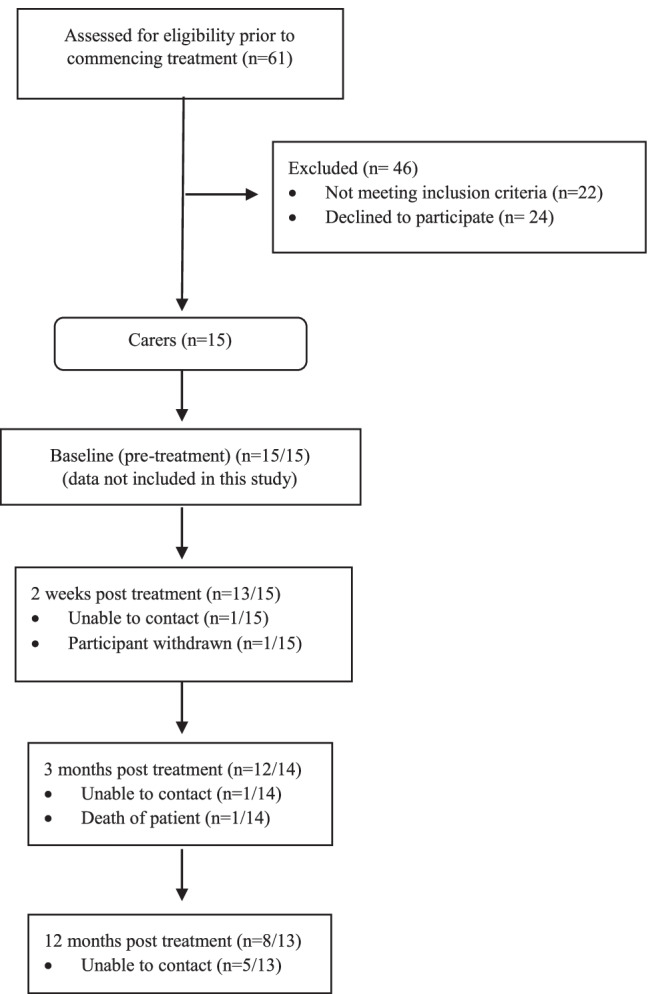
Participant recruitment and retention

Fourteen carers were included in this study. Diagram 1 outlines participant recruitment and retention to the study. Carer demographics are outlined in Table 1. Five carers had experience supporting patients needing gastrostomy tube placement to support their nutrition intake (4 carers of patients receiving chemoradiation and 1 carer of a patient having surgery and PORT) Fig. 1.

**Table 1:** Participant demographics

Participant ID	Sex	Relationship to patient	Patient diagnosis	Usual place of residence	Nutrition support	Treatment modality
C1	Female	Daughter	Oral cavity SCC	Regional	NGT and Oral	Surgery
C2	Female	Wife	Laryngeal SCC	Urban	Oral	Surgery
C3	Female	Wife	Oral cavity SCC	Urban	Oral	Surgery
C4	Female	Sister	Oral cavity SCC	Rural	NGT and Oral	Surgery + PORT
C5	Female	Wife	Nasopharyngeal SCC	Urban	NGT and Oral	Surgery + PORT
C6	Female	Sister	Oropharyngeal SCC	Regional	NGT and Oral	Surgery + PORT
C7	Female	Daughter	Oral cavity SCC	Regional	NGT and Oral	Surgery + PORT
C8	Female	Wife	Oral cavity SCC	Urban	Oral	Surgery + PORT
C9	Female	Mother	Oral cavity SCC	Regional	Gastrostomy	Surgery + PORT
C10	Female	Wife	Oropharyngeal SCC	Regional	Gastrostomy	(Chemo)radiation
C11	Female	Wife	Oral cavity SCC	Urban	Gastrostomy	(Chemo)radiation
C12	Male	Husband	Oropharyngeal SCC	Urban	Oral	(Chemo)radiation
C13	Female	Wife	Nasopharyngeal SCC	Urban	Gastrostomy	(Chemo)radiation
C14	Female	Wife	Oropharyngeal SCC	Urban	Gastrostomy	(Chemo)radiation

*PORT* = Post-operative radiotherapy

*SCC* = Squamous cell carcinoma

*NGT* = Nasogastric tube

### Themes

Two main themes were identified across each treatment modality. These included (1) access to information and support from healthcare professionals and (2) adjustment to the physical and psychological impact of treatment. Within each theme, subthemes were identified; these are presented in the text in italics and outlined in Table 2 along with representative quotes.

**Table 2:** Quotes related to each main theme

Theme	Treatment	Subtheme	Representative quotes
Access to information and support from healthcare professionals	Radiation	Easy access to information and support	“Well, I’ve got a lot of information from dietitians at the hospital and recipe books and that list was sent home with all the different proteins and fats was very helpful” (C6, 2 weeks post-treatment) “I certainly was aware of a lot of things after the multidisciplinary meeting that was put on. [patient name] had an appointment at the time, but I went along” (C5, 2 weeks post-treatment) “If we felt we needed to see somebody we put it in motion through the doctors we were seeing or whoever and the clinicians they would put in the recommendation and that’s been awesome and I think it’s one of those things you really need to push with people that all the things that are available just to realise what a big impact they make on the lives of people when they go through radiation and the effects” (C8, 3 months post-treatment) “I’ve got the hospital number here or if I can’t hold of somebody here, I’m sure somebody down in Brisbane would help me out if I had to…” (C10, 3 months post-treatment)
		Looking to the internet for new ideas	“I have done a lot of work [looking online] I just try to change everything all the time and I do that also not only for his nutrition but to get his taste buds going again as well” (C11, 3 months post-treatment)
	Surgery	Trial and error	“Um really we weren’t told what he could and couldn’t eat so we just had to work out what he felt comfortable eating” (C2, 2 weeks post-treatment)
		Reliant on the patient to provide information	“Yeah, looking at what [patient name] is prepared to tell me rather than what the hospital wants and trying my best to come up with things that he is prepared to eat that don’t sting or hurt or make things worse” (C2, 2 weeks post-treatment)
		Information specific to carers	“I would suggest that the patient in the hospital be given the package of information from the patient saying you’ve had the surgery, you’re here for 8 days, sometime in those 8 days do up a diet plan, put it in writing, give it to the patient where you’ve got access to them readily and say at the top of it ‘support person information’. That way the patient can read it and go this is what I need to do and then they can give it to the support person who can read it and go ah this is what I need to do” (C3, 3 months post-treatment) “They could tell us to go on the internet, to this site if you have access to a computer, and I know some people won’t but go on this website and have a look, this is what you need to be aware of. It doesn’t have to be in writing but being an old gal that’s what I’d prefer” (C3, 3 months post-treatment)
Adjustment to the physical and psychological impact of treatment	Radiation	Something you can rely on	“…he accepted it really quickly [gastrostomy tube] and I think once he knew that it was going to be there as a support system for him, he welcomed it, necessary actually we found it to be a necessity” (C11, 2 weeks post-treatment)
		Frustration	“It’s definitely a different lifestyle that’s for sure” (C10, 3 months post-treatment) “I think personally he could have kept up with a little bit of extra nutrition with that Fortisip for just a little bit longer, but he was racing to feel normal, and I think if we looked at it back again, I would have kept it [gastrostomy tube] in there for a little bit longer” (C11, 3 months post-treatment)
		Food aversion	“…difficult for the kids like I don’t um I worry that him not eating… the kids get a bad feeling on food so we are always talking about why he’s not eating and you know the reasons behind it” (C13, 12 months post-treatment)
		Trying to lead a healthier lifestyle	“It’s completely flipped our lifestyle… you’ve got to get out and make memories… things that used to worry us, don’t worry us as much anymore” (C14, 12 months post-treatment) “Now we are out of the treatment period, it’s about keeping [patient name] healthy” (C13, 12 months post-treatment)
		Fatigue and burnout	“I’m sick of telling him what to do” (C7, 3 months post-treatment) “We were committing to something [treatment] without really knowing what we were committing to” (C7, 3 months post-treatment)
	Surgery	Confronting	“The way she looks, it’s just been a shock and we are all still waiting for the swelling to go down. It’s been very confrontational” (C1, two weeks post-treatment) “It’s been a whole new thing with how he looks because his mouth is narrow, so he looks different” (C2, three months post-treatment)
		Wondered if it was worth it	“I just feel there could have been a more balanced approach to it because sometimes it should be more about quality of life because now his quality of life is significantly poor and compared to if he had not had the operation I don’t know, he would have certainly died there’s no doubt about that…” (C7, 3 months post-treatment)
		Distressing seeing other people’s reactions	“I can’t explain it, but people think that something is mentally wrong with him. So, he gets very angry and frustrated in those situations of course. But that doesn’t help people are judging them not just for how they look but their mental capacity as well” (C7, 3 months post-treatment) “…because of his mouth food sometimes drops down the front with the shape of the mouth being different now” (C2, 3 months post-treatment)

### Access to information and support from healthcare professionals

#### Radiation

*Easy access to information* *and support* from healthcare professionals was described by carers of patients throughout radiation treatment (“yes, everyone here has explained a lot and shown me a lot from the dietitians right through to the speech people” C10, 2 weeks post-treatment). Having weekly scheduled appointments with the dietitian and speech pathologist provided the carer with the opportunity to plan for and attend appointments to find out more information on the patient’s nutrition care needs. Overall, carers emphasised the importance of attending the HNC talk, where they were provided with information from a range of healthcare professionals within the multidisciplinary team including how they could access support in those areas if needed (“…it was very helpful for me” C5, 2 weeks post-treatment). Knowing support was easily accessible meant that carers who were unable to attend scheduled appointments felt they could contact healthcare professionals at another time to address any concerns they had (“If we felt we needed to see somebody we put it in motion through the doctors we were seeing or whoever and the clinicians they would put in the recommendation and that’s been awesome” C8, 3 months post-treatment).

Throughout the treatment period, some carers spoke about avoiding looking for information on the Internet, both due to feeling satisfied with the information and support they were provided and to keep things “really simple for ourselves” (C11, 2 weeks post-treatment). Three months after treatment completion, when less frequent support was available, some carers spoke about *looking to the internet for new ideas* on how to support the patient with their nutrition intake (“I’ve learnt that through the internet” C11, 3 months post-treatment). Carers expressed that this period was challenging for them as it was also tied to the same time many patients were getting their gastrostomy tube removed as they returned to managing oral intake (“I just felt it was a bit tougher for me” C11, 3 months post-treatment). Despite these challenges, at 3 and 12 months post-treatment completion, carers remained confident that information and support from healthcare professionals was accessible if needed.

#### Surgery

Carers of patients having surgery strongly expressed how challenging it was to access support from healthcare professionals on the hospital ward to provide them with information on the patient’s nutritional needs post-surgery. Consequently, carers spoke about trying to work out what foods and fluids the patient should be having through *trial and error.* This included searching for information online about what foods they should be preparing for the patient after their surgery (“I did look at some nutrition things online” C2, 2 weeks post-treatment).

Several carers spoke about being *reliant on the patient to provide information* about their supportive care needs post-surgery. One carer felt confident with the types of foods she could prepare after her mother brought home written information about the texture modified diet she was prescribed post-surgery (“she was just given sheets from the hospital, and she seems to be eating as much blended stuff as she can” C1, 2 weeks post-treatment). In contrast, one carer described her frustration in not having any information to refer to and feeling her partner was not providing her with the right information.

Where appointments with the dietitian and speech pathologist were scheduled for the patient after surgery, some carers found it difficult to attend due to other commitments. Acknowledging how challenging it was to access information and support both on the hospital ward and in the clinic setting, some carers conveyed the need for *information specific to carers* as a separate resource to the information the patient is provided (“I should have got something saying do this to help, to give some direction” C3, 3 months post-treatment). This included carers speaking about the need to be referred to information online to provide a way to access information and support as needed in the home environment.

### Adjustment to the physical and psychological impact of treatment

#### Radiation

Five out of 11 carers supported patients with a gastrostomy tube throughout their radiation treatment. Two weeks after treatment completion, carers reflected on the gastrostomy tube as being *something you can rely on* when eating and drinking became more difficult (“I haven’t had to do a whole lot with his diet because he’s having liquid through the PEG” C10, 2 weeks post-treatment).

Three months post-treatment completion, some carers described a level of *frustration* in no longer having the security of the gastrostomy tube to help support the patient with their nutrition intake. However, carers spoke about the impact the gastrostomy tube had on their social interactions with others due to the time taken to fit in tube feeds (“because of feeding it makes it hard to do anything” C10, 3 months post-treatment) and feeling guilty in both leaving the patient at home or eating and drinking in front of them (“I feel that guilty feeling of going out knowing that he’s at home” C13, 12 months post-treatment).

One-year post-treatment completion, most carers of patients who were treated with chemoradiotherapy spoke about feeling as though the weight of trying to support the patient with their nutrition intake was significantly eased. However, one carer described trying to protect her children from developing an unhealthy relationship with food after seeing their father continue to struggle to eat and drink due to *food aversion* as a result of the chronic side effects of his radiation treatment (“I mean they are kids, they are influential and I want them to see food as a healthy thing and a good thing” C13, 12 months post-treatment). Overall carers spoke positively about the future, highlighting that their focus now was on *trying to lead a healthier lifestyle*. For some this included taking better care their own and the patients mental health (“we try not to sweat the small stuff anymore” C14, 12 months post-treatment), as well as the physical health of the patient by trying to maintain weight in a healthier weight range (“…he’s trying to stay that weight, he eats healthy and we work to keep our portions a bit smaller” C14, 12 months post-treatment).

In contrast, carers of patients treated with surgery and PORT did not express the same sentiment as they looked forward to the future, instead expressing fatigue and burnout in their supportive care role (“I feel a bit overwhelmed actually” C5, 2 weeks post-treatment, “I wasn’t supported when he was going through the treatment and that is partially why I was so burnt out” C7, 12 months post-treatment).

#### Surgery

Carers of patients having surgery described how *confronting* it was to see changes in their loved one’s appearance after surgery. In addition to swelling and bruising after surgery, seeing lines, tubes for feeding and breathing and wiring attached to the patient on the hospital ward further amplified this response (“I don’t know, I don’t think I expected anything because I didn’t know what to expect, I never expected her to look like an octopus” C1, 2 weeks post-treatment).

Understanding that her father would have died without treatment, one carer *wondered if it was worth it* after seeing his quality of life rapidly deteriorate due to the extent of disfigurement after surgery (“…he’s lost half his face and looks terrible, and now he can’t enjoy his life either so it’s significant” C7, 3 months post-treatment). Some carers expressed how *distressing it was to see other* *people’s* *reactions* to the impact of the surgery on their loved one. One carer described how her father was treated as though he has a mental disability due to the difficulty he had speaking (“people treat him like he’s got a mental impairment and he doesn’t” C7, 12 months post-treatment). To avoid being seen by others, one carer avoided social events due to the functional changes her partner experienced impacting his eating and drinking (“he doesn’t like people watching him eat” C2, 3 months post-treatment). Contributing further to this was the fear that the ongoing management of physical symptoms post-surgery was perceived to be a sign that the patient was not getting better (“…my fear since April is that he will die” C3, 12 months post-treatment).

## Discussion

Differences in carer experiences of nutrition care throughout surgery and radiation treatment for HNC have not been previously explored. With increasing recognition of the crucial role carers play in supporting patients through the physical and psychological impact of the disease and treatment [8], it is paramount that we understand areas where more support is needed to ensure provision of best practice care and optimise outcomes for both the patient and carer. Therefore, the aim of this study was to explore differences in carer specific needs regarding the provision of nutrition support throughout surgery and radiation/chemoradiation treatment and extending into the survivorship period.

Information and support from healthcare professionals is a critical component of cancer care, yet remains an unmet need in informal carers as seen in this study and others [12, 20]. Although not specific to HNC, up to 39% of carers of patients with cancer are unsatisfied with the amount of time spent with healthcare professionals to address their information and support needs [12]. Higher levels of carer needs are directly correlated to a limited understanding of the healthcare system, including awareness of support options available [20]. Carers of patients treated with radiation expressed a lower level of care needs in comparison to carers of patients treated with surgery. Having scheduled appointment times each week with the dietitian and speech pathologist throughout radiation, and awareness of information and support options available from the multidisciplinary team presented prior to radiation starting, provided carers with an understanding of the health system and services available to them. Findings of an Australian study evaluating the effectiveness of weekly appointments with the dietitian and speech pathologist throughout radiation treatment for HNC found that where clinicians had identified that weekly nutrition and swallowing support was not indicated, some patients expressed a desire to keep their appointment for more general support [21]. Similarly, carers in our study did not delineate their supportive care needs to individual healthcare professionals within the multidisciplinary team, but rather valued knowing that support was easily accessible, with scheduled appointments with the dietitian and speech pathologist providing a means to access information and support across the multidisciplinary team.

In contrast, without having the support offered to patients having radiation treatment, carers of patients having surgery expressed a lack of awareness of support options available to them. Studies have shown that carers of patients in acute hospital care often feel unprepared when the patient is discharged home because they do not have enough information and support from healthcare professionals to assist them in their supportive care role [22]. This period of time is very stressful for carers due to many patients having high care needs after surgery and adjustment to taking on the carer role [20]. In the acute care setting, healthcare professionals do not always recognise that family members need individualised guidance that considers their specific needs in the caring role [23]. Involving family members in the discharge process can both support carer self-efficacy and promote the outcomes of the patient’s recovery [24].

Using a longitudinal study design provided insight into how information and support needs of carers vary as patients progress through the treatment trajectory. Carers of patients treated with radiation described the period immediately after treatment completion the most challenging when the frequency of support was no longer available. This also included a transition away from tube feeding to oral intake to meet nutritional requirements. Preparing food and drinks to support the patient with their nutrition intake was a role and responsibility many carers took on themselves, and in this period, many carers turned to the Internet for ideas on what foods to prepare taking into consideration the side effects of treatment including dysgeusia and xerostomia. Previous studies have reported the challenges carers experience in supporting the patient with everyday activities including meal preparation and the negative impact this has on their own wellbeing including for some, social isolation [13]. Further away from treatment completion, carers of patients treated with radiation who had not had surgery prior expressed lower care needs as their goals were shifted to looking forward to the future. This highlights a need to ensure information, and support is accessible immediately after radiation treatment including referral by healthcare professionals to information online that addresses the practicalities of nutrition support including the transition from tube feeding to oral intake and meal ideas, as well as ensuring carers can access support to address their psychosocial concerns.

Carers of patients treated with surgery expressed high care needs that were unmet at each interview timepoint. A study by Beaver et al. found that patients are the greatest information source for carers [25]. However, our study found that many carers felt they could not rely on information provided to them filtered through the patient’s lens. Immediately after treatment, where written information was not provided, some carers feared they would harm the patient or do damage to the surgical site with the types of foods and drinks they were preparing for the patient. Contributing to the psychological distress many carers expressed was the impact of the surgery on the patient’s appearance. Many patients find themselves unprepared for changes in their appearance after surgical treatment for head and neck cancer, and many experience distress seeing the reactions of others including their family and friends [26]. Similarly, our study found that carers felt unprepared for the severity of the changes to the patient’s appearance and distress themselves seeing other people’s reactions. Consequently, many carers also become socially isolated in this period. While follow-up care is provided in the outpatient setting with the dietitian and speech pathologist, some carers in our study did not attend these appointments. Similarly, where weekly support was available through radiation treatment for patients needing post-operative radiation, some carers also did not attend. This strongly suggests that neglecting to engage with carers on the hospital ward after surgery may leave them feeling unsupported and disengage them from accessing information and support later in the treatment trajectory even when this is made more accessible.

Programs and support for carers are not embedded within cancer care services [27]. Furthermore, there is little guidance for healthcare professionals on ways they meet the needs of carers [27]. A study by Roen et al. found that use of a caregiver screening tool provided the opportunity to tailor consultations to the information and support needs of both the patient and carer [28]. However, limited resources and fearing carer involvement would add extra work to an already heavy workload were barriers reported by healthcare professionals to implementing carer support into practice [28]. This highlights the need for changes within healthcare organisations including education in carer support and development of standardised care pathways.

A recent study by Wishart et al. found that where carers were provided opportunities to access extra support, fewer than 5% requested a referral to healthcare professionals to discuss their concerns [29]. Sadly, many carers consider their psychosocial distress to be normal and feel that available resources should be directed at supporting the patient [29]. Carers in our study turned to the internet to seek out information when their information needs were not met. Studies have shown that online interventions provide carers with the opportunity to access information and support in their own time and at their own pace, demonstrating improvements in knowledge and communication [30]. Further exploration and development of online resources for carers provides a means of creating access to information and support outside of the hospital environment as a step towards ensuring they feel supported in their supportive care role, regardless of treatment modality.

### Strengths and limitations

Capturing the carer experience at three separate time points provided a unique insight into the changing information and support needs throughout the treatment and survivorship period. However, interpretation of the findings must take into consideration the limitations of the study. As interviews were conducted at time points after treatment completion, this meant that carers of patients treated with surgery and PORT were more likely to report on their most recent experience of radiation and may have omitted important information about their experience in supporting the patient through their surgery as it no longer was at the forefront of their mind. Furthermore, this study included a higher ratio of female to male carers (13:1), limiting the exploration of experiences of male carers. It is also important to acknowledge that not all cancer care centres may provide weekly evaluation by the speech pathologist and dietitian throughout radiation treatment, therefore limiting the generalisability of these results. Using hermeneutic inquiry, the intent of the study was to provide a description of the experience of carers supporting patients with their nutrition care needs throughout HNC treatment and survivorship, rather than generalise the findings. However, a limitation of this study was the lack of demographic information collected to understand how the experience of carers in this study vary by factors including socioeconomic status, cultural background and health literacy levels.

### Conclusion

This study highlights the increasing need to ensure carers are included in information and support provided to patients throughout their treatment trajectory. Having structured support available to patients and carers throughout radiation treatment meant that carer needs were reduced. However, without the opportunity for structured support in the inpatient setting, many carers expressed high care needs in supporting patients in the post-surgical phase, extending into the survivorship period.

## Supplementary Information

Below is the link to the electronic supplementary material.

Supplementary file1 (DOCX 21 KB)

## Data Availability

The datasets generated and analysed during the current study are available from the corresponding author on reasonable request.

## References

[1] Bray F, Ferlay J, Soerjomataram I, Siegel R, Torre L, Jemal A (2018). Global cancer statistics 2018: GLOBOCAN estimates of incidence and mortality worldwide for 36 cancers in 185 countries. CA Cancer J Clin.

[2] Ferlay J, Colombet M, Soerjomataram I, Mathers C, Parkin D, Pineros M, Znaor A, Bray F (2019). Estimating the global cancer incidence and mortality in 2018: GLOBOCAN sources and methods. Int J Cancer.

[3] Marur S, Forastiere A (2008). Head and neck cancer: changing epidemiology, diagnosis, and treatment. Mayo Clin Proc.

[4] Bressan V, Bagnasco A, Aleo G, Catania G, Zanini P, Timmins F, Sasso L (2017). The life experience of nutrition impact symptoms during treatment for head and neck cancer patients: a systematic review and meta-synthesis. Support Care Cancer.

[5] Britton B, Baker A, Clover K, McElduff P, Wratten C, Carter G (2017). Heads up: a pilot trial of a psychological intervention to improve nutrition in head and neck cancer patients undergoing radiotherapy. Eur J Cancer Care.

[6] Findlay M, Bauer J, Brown T (2011) Evidence-based practice guidelines for the nutritional management of adult patients with head and neck cancer. Sydney. Cancer Council Australia. http://wiki.cancer.org.au/australia/COSA:Head_and_neck_cancer_nutrition_guidelines. Accessed 2021 May 6

[7] Sun V, Raz J, Kim J (2019). Caring for the informal cancer caregiver. Curr Opin Support Palliat Care.

[8] Halkett G, Golding R, Langbecker D, White R, Jackson M, Kernutt E, O'Connor M (2020). From the carer’s mouth: a phenomenological exploration of carer experiences with head and neck cancer patients. Psychooncology.

[9] Mayre-Chilton K, Talwar B, Goff L (2011). Different experiences and perspectives between head and neck cancer patients and their care-givers on their daily impact of a gastrostomy tube. J Hum Nutr Diet.

[10] Kam D, Salib A, Gorgy G (2015). Incidence of suicide in patients with head and neck cancer. JAMA Otolaryngol-Head Neck Surg.

[11] Castellanos E, Dietrich M, Bond S, Wells N, Schumacher K, Ganti A, Murphy B (2019). Impact of patient symptoms and caregiving tasks on psychological distress in caregivers for head and neck cancer (HNC). Psychooncology.

[12] Hanly P, Maguire R, Balfe M, Hyland P, Timmons A, O'Sullivan E, Butow P, Sharp L (2016). Burden and happiness in head and neck cancer carers: the role of supportive care needs. Support Care Cancer.

[13] Nund R, Ward E, Scarinci N, Cartmill B, Kuipers P, Porceddu S (2014). Carers’ experiences of dysphagia in people treated for head and neck cancer: a qualitative study. Dysphagia.

[14] Patterson J, Rapley T, Carding P, Wilson J, McColl E (2013). Head and neck cancer and dysphagia; caring for carers. Psychooncology.

[15] Hiatt J, Young A, Brown T, Banks M, Bauer J (2021). Patient and carer experience of nutrition care throughout and beyond treatment for head and neck cancer: a qualitative longitudinal study. Supportive Care in Cancer.

[16] Braun V, Clarke V (2019). Reflecting on reflexive thematic analysis. Qual Res Sport Exerc Health.

[17] Koch T (1996) Implementation of a hermeneutic inquiry in nursing: philosophy, rigour and representation. 10.1046/j.1365-2648.1996.17224.x 10.1046/j.1365-2648.1996.17224.x 8807394

[18] Molassiotis A, Rogers M (2012). Symptom experience and regaining normality in the first year following a diagnosis of head and neck cancer: a qualitative longitudinal study. Palliat Support Care.

[19] Clarke V, Braun V (2014). Thematic analysis. J Posit Psychol.

[20] Chen S, Lai Y, Liao C, Huang B, Lin C, Fan K, Chang J (2014). Unmet supportive care needs and characteristics of family caregivers of patients with oral cancer after surgery. Psychooncology.

[21] Wall L, Cartmill B, Ward E, Hill A, Isenring E, Porceddu S (2016). Evaluation of a weekly speech pathology/dietetic service model for providing supportive care intervention to head and neck cancer patients and their carers during (chemo) radiotherapy. Support Care Cancer.

[22] Abrahamson V, Jensen J, Springett K, Sakel M (2017). Experiences of patients with traumatic brain injury and their carers during transition from in-patient rehabilitation to the community: a qualitative study. Disabil Rehabil.

[23] Roscigno C (2016). Parent perceptions of how nurse encounters can provide caring support for the family in early acute care following children’s severe traumatic brain injury. J Neurosci Nurs.

[24] Mitchell S, Laurens V, Weigel G, Hirschman K, Scott A, Nguyen H, Howard J, Laird L, Levine C, Davis T, Gass B, Shaid E, Li J, Williams M, Jack B (2018). Care transitions from patient and caregiver perspectives. Ann Fam Med.

[25] Beaver K, Witham G (2007). Information needs of the informal carers of women treated for breast cancer. Eur J Oncol Nurs.

[26] Gibson C, O'Connor M, White R, Jackson M, Baxi S, Halkett G (2021). ‘I didn’t even recognise myself’: survivors’ experiences of altered appearance and body image distress during and after treatment for head and neck cancer. Cancers.

[27] Kim Y, Carver C (2019). Unmet needs of family cancer caregivers predict quality of life in long-term cancer survivorship. J Cancer Surviv.

[28] Røen I, Stifoss-Hanssen H, Grande G, Kaasa S, Sand K, Knudsen A (2019). Supporting carers: health care professionals in need of system improvements and education-a qualitative study. BMC Palliat Care.

[29] Wishart L, Brown B, Nund R, Fotinos E, Hutchison A, Ward E, Porceddu S (2021). A prospective study monitoring carer distress during (chemo) radiotherapy for head and neck cancer via an electronic platform. J Med Radiat Sci.

[30] Heynsbergh N, Heckel L, Botti M, Livingston P (2018). Feasibility, useability and acceptability of technology-based interventions for informal cancer carers: a systematic review. BMC Cancer.

